# Idiopathic intracranial hypertension: an illustrated guide for the trainee radiologist

**DOI:** 10.1590/0100-3984.2021.0091-en

**Published:** 2022

**Authors:** Luísa Becker Savastano, Juliana Ávila Duarte, Thiago Bezerra, José Thiago de Souza Castro, Mariana Dalaqua, Fabiano Reis

**Affiliations:** 1 Department of Radiology, Universidade Estadual de Campinas (Unicamp), Campinas, SP, Brazil; 2 Department of Radiology and Diagnostic Imaging, Hospital de Clínicas de Porto Alegre (HCPA), Porto Alegre, RS, Brazil; 3 Hôpitaux Universitaires de Genève, Service de Radiologie, Geneva, Switzerland

**Keywords:** Pseudotumor cerebri, Neuroimaging, Papilledema, Hipertensão intracraniana idiopática, Neuroimagem, Papiledema

## Abstract

Idiopathic intracranial hypertension is characterized by increased intracranial pressure, headache, and visual perturbations. Although the pathophysiology of idiopathic intracranial hypertension is obscure, several mechanisms have been proposed, such as increased cerebral blood volume, excessive cerebrospinal fluid volume (due to high production or impaired resorption), and inflammatory mechanisms as a likely cause of or contributor to impaired cerebrospinal fluid circulation. It predominantly affects women of reproductive age who are overweight or obese. The most common symptoms are daily headache, synchronous pulsatile tinnitus, transient visual perturbations, and papilledema with visual loss. The main neuroimaging findings are a partially empty sella turcica; flattening of the posterior sclera; transverse sinus stenosis (bilateral or in the dominant sinus); a prominent perioptic subarachnoid space, with or without optic nerve tortuosity; and intraocular protrusion of the optic nerve head. The main complication of idiopathic intracranial hypertension is visual loss. Within this context, neuroimaging is a crucial diagnostic tool, because the pathology can be reversed if properly recognized and treated early.

## Abstract (pt)

A hipertensão intracraniana idiopática é caracterizada por aumento da pressão intracraniana, cefaleia e manifestações visuais. Apresenta fisiopatologia incerta, porém, alguns mecanismos já foram propostos, como o aumento do volume sanguíneo cerebral, o excesso de líquor por aumento da produção ou a redução da reabsorção, e mecanismos inflamatórios como fator causal ou mesmo determinando limitação na circulação do líquor. Predomina em mulheres obesas em idade reprodutiva. Os sintomas e sinais mais comuns são cefaleia diária, zumbido síncrono ao pulso, obscurecimentos visuais transitórios e papiledema com perda visual. Os principais achados em neuroimagem são: sela turca vazia, achatamento posterior do globo ocular/esclera, estenose do seio transverso bilateral ou do seio dominante, distensão do espaço liquórico perióptico com ou sem tortuosidade do nervo óptico e protrusão intraocular da cabeça do nervo óptico. A principal complicação da hipertensão intracraniana idiopática é a perda visual. Nesse contexto, o papel da neuroimagem no diagnóstico é fundamental, pois a doença pode ser revertida se devidamente reconhecida e precocemente tratada.

## INTRODUCTION

Idiopathic intracranial hypertension (IIH), previously known as benign intracranial hypertension or pseudotumor cerebri, is a syndrome characterized by increased intracranial pressure, the cause of which has not been fully elucidated. In individuals with IIH, the results of the neurological examination and cerebrospinal fluid (CSF) analysis can be nearly normal, except for signs and symptoms such as headache, papilledema, tinnitus, cranial nerve palsy, and visual loss. Although some authors have suggested that IIH without papilledema constitutes an atypical variant^(1)^, Toscano et al.^(2)^ concluded that, despite the low (5%) prevalence in their patient sample, papilledema should be considered a specific sign of IIH. The mechanisms proposed include increased cerebral blood volume, excess CSF due to increased production or reduced reabsorption, and inflammatory processes.

Corroborating the idea of an inflammatory mechanism, astrocyte-targeted autoimmunity has been documented, as has the presence of oligoclonal bands, a marker of intrathecal inflammation, which is seen in approximately one third of all patients with IIH. Antibodies against glial fibrillary acidic protein have been detected in the serum of patients with active-phase IIH^(3)^. Lymphatic system dysfunction could also play a role in the pathophysiology of IIH, because of interstitial fluid overload. That can be attributed to loss of aquaporin 4 or an increase in the permeability of the blood-brain barrier during perivascular inflammation, which results in expansion of the interstitial compartment^(2)^.

Magnetic resonance imaging (MRI) and venography by MRI or venous MR angiography (MRA) are essential for the diagnosis of IIH, because they allow the neuroimaging aspects of the syndrome to be characterized, as well as allowing other structural causes of increased intracranial pressure, especially cerebral venous thrombosis, to be excluded. The diagnosis of IIH is based on the identification of at least three characteristic neuroimaging findings-empty sella turcica; posterior flattening of the globe/sclera; transverse sinus stenosis (bilateral or of the dominant sinus); or distention of the perioptic CSF space, with or without tortuosity of the optic nerve-those changes being especially important in patients who do not present with papilledema^(1)^.

## EPIDEMIOLOGY AND PREDISPOSING FACTORS

The population most affected by IIH is that of young women who are classified as overweight or obese (body mass index > 25 kg/m^2^), the reported incidence being 19 cases/100,000 in that population. Among such patients, 68-98% present with headache. Other clinical features include pain, pulsatile tinnitus, and visual disturbances that can lead to blindness^(4)^.

Other illnesses and the use of medications can also trigger IIH. For those cases, the term “idiopathic” should be avoided and the findings of intracranial hypertension should be considered to result from the factors in question, all of which all merit investigation in patients in whom the imaging findings are suggestive of IIH^(2)^.

## CLINICAL MANIFESTATIONS

Symptoms of IIH include headache that worsens on lying down, diplopia, transient visual obscuration, scotomas, pulsatile tinnitus, and vertigo, as well as retro-orbital, cervical, or facial pain. The most common manifestations are a loss of peripheral visual acuity and paresis of the lateral rectus muscle due to involvement of the abducens nerve, as well as bilateral papilledema, which can be symmetrical or asymmetrical. Of all patients with IIH, 10% develop optic nerve atrophy and severe visual impairment due to the prolonged course of papilledema, and some become blind. Therefore, the syndrome requires immediate detection and action, which can often prevent visual loss^(2)^.

Some patients with IIH may be asymptomatic, whereas others can have intracranial hypotension or CSF otorrhea/rhinorrhea, which, although rare, are suggestive of IIH. The last condition can occur when chronically high intracranial pressure causes CSF leakage due to remodeling of the skull base, mainly involving the ethmoidal trabeculae and the lateral sphenoid recesses, accompanied by meningoencephaloceles. Such cases are diagnosed mainly when the symptoms of IIH occur after surgical repair, leading to a new increase in intracranial pressure, or when a CSF leak occurs after surgery.

## DIAGNOSIS

**Figure 1:**
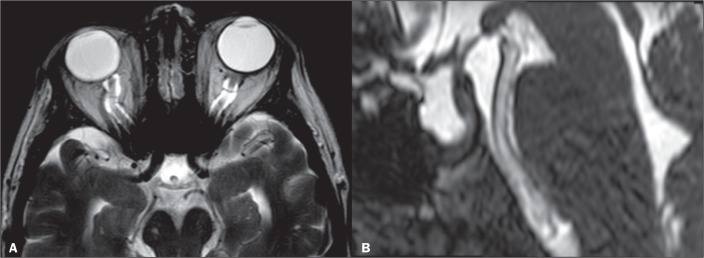
Axial and sagittal T2-weighted MRI scans (A and B, respectively) showing distention of the CSF sheath of the optic nerves, slight mild optic nerve tortuosity, flattening of the posterior sclera and an empty sella turcica.

**Figure 2:**
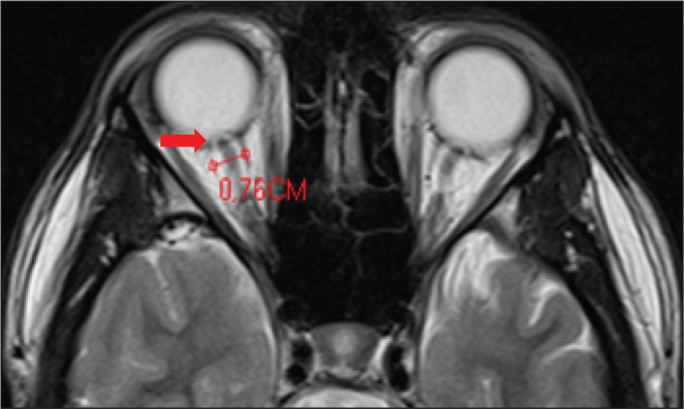
Axial T2-weighted MRI scan showing an increase in the diameter of the optic nerve sheath (5 mm behind the papilla can be considered the upper limit of normal). Note the intraocular protrusion of the optic nerve head.

The diagnostic criteria for IIH are based on clinical signs and symptoms, as well as on MRI findings and the measurement of CSF pressure by lumbar puncture. Most radiological findings of IIH have high specificity but low sensitivity. As illustrated in Figure 1, flattening of the posterior sclera is, in isolation, the most specific finding^(5)^. Flattening of the posterior sclera and an empty sella were the most common findings in the study conducted by Brodsky et al.^(6)^. Other MRI findings of IIH, as shown in Figure 2, include distention of the CSF sheath surrounding the optic nerve, nerve tortuosity, intraocular protrusion of the optic nerve head, and posterior flattening of the globe^(5)^.

**Figure 3:**
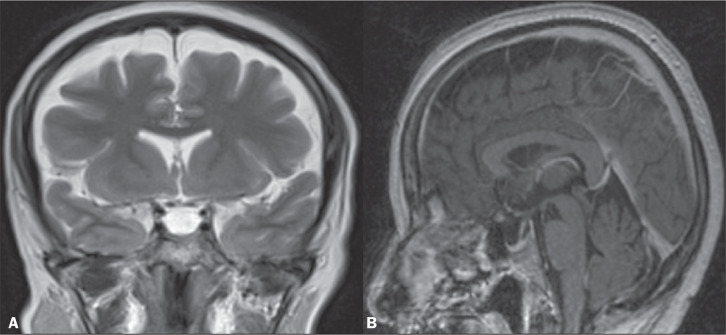
Coronal T2-weighted MRI scan (A) and contrast-enhanced sagittal T1-weighted MRI scan (B) showing a partially empty sella turcica, with a distended stalk. Note the slight reduction in the dimensions of the third ventricle and the lateral ventricles.

**Figure 4:**
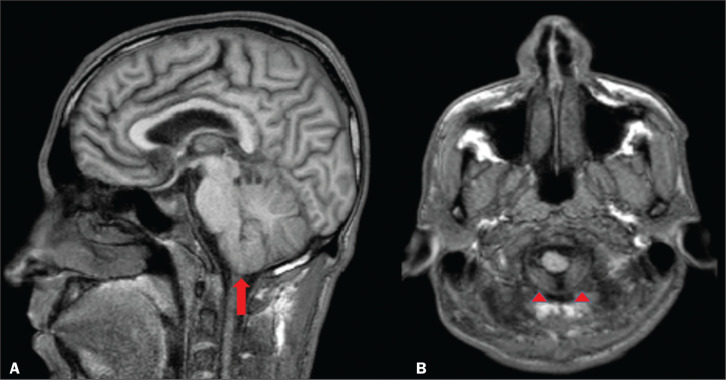
Sagittal and axial T1-weighted MRI scans (A and B, respectively) showing mild inferior insinuation of the cerebellar tonsils through the foramen magnum (arrow in A; arrowheads in B).

In cases of IIH, the ventricles can be small^(5)^, as depicted in Figure 3. Inferior displacement of the cerebellar tonsils (Figure 4), mimicking a Chiari I malformation, can be observed. It is important to differentiate IIH from changes related to a Chiari I malformation (in which tonsillar ectopia is usually more pronounced), given that the two entities are treated differently^(7)^.

**Figure 5:**
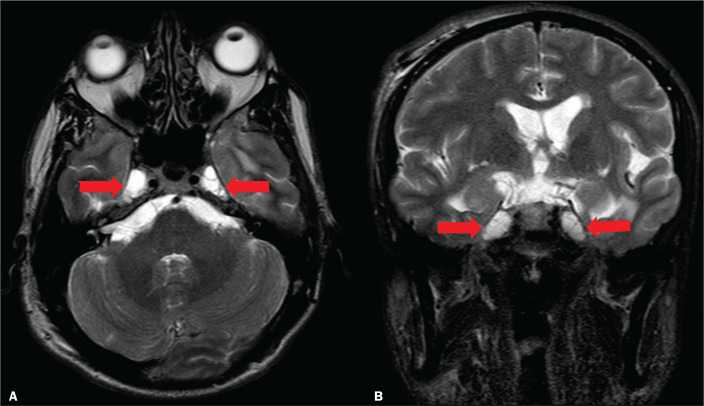
Axial and coronal T2-weighted MRI scans (A and B, respectively) showing ectasia of Meckel’s cave (arrows), an alteration that can be observed in the context of IIH. As unrelated findings, there are postoperative changes in the extracranial tissues of the right frontal convexity and in the signal of the left temporal periventricular region, together with a small cortico-subcortical area of ischemia as a sequela in the periphery of the right cerebellar hemisphere.

On MRI, it is also possible to observe widening of Meckel’s cave (Figure 5), prominent arachnoid granulations, and petrous apex meningocele. A meningocele is mechanically similar to the empty sella and increases the volume of the subarachnoid space, thus mitigating the increase in intracranial pressure^(5)^.

**Figure 6:**
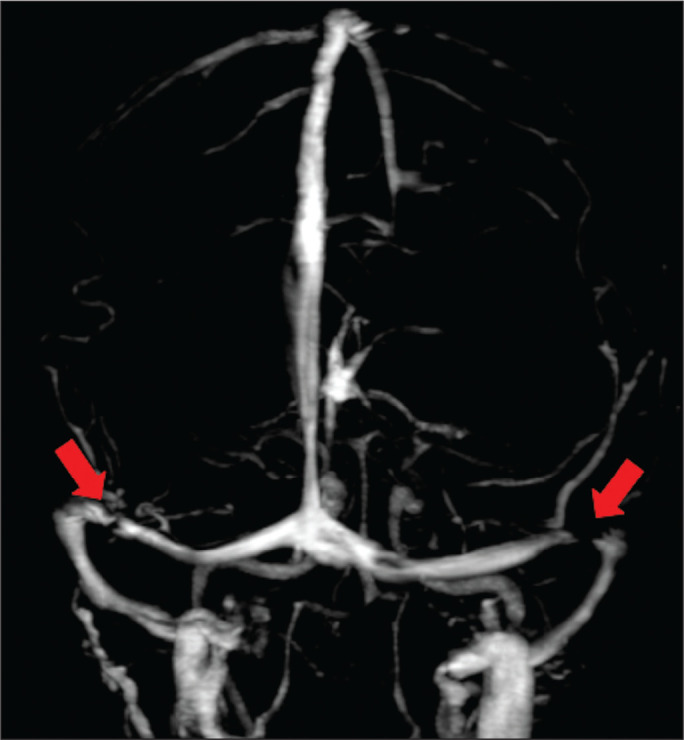
Three-dimensional venous MRA reconstruction with maximum intensity projection showing bilateral transverse sinus stenosis (arrows).

In patients with other IIH findings, a diagnosis of cerebral venous thrombosis can be excluded through the use of MRI, ideally with venous MRA. It also allows the identification of dural sinus stenoses (Figure 6) or of a prominent occipital emissary vein, which has been described as a contributor to collateral venous flow in cases of IIH secondary to dural sinus stenosis^(8)^. Abducens nerve palsy is seen in 12% of adults and in 9-48% of children. Less frequently, there can be palsy of other cranial nerves, including the oculomotor, trochlear, facial, glossopharyngeal, and hypoglossal nerves^(9)^.

Acetazolamide, a carbonic anhydrase inhibitor, can be used in order to reduce the production of CSF by the choroid plexus. The expected effects of acetazolamide administration are improved vision and reduction of the papilledema^(2)^. Other therapeutic approaches include weight loss, lumbar puncture, and treatment with topiramate or furosemide, as well as optic nerve sheath fenestration in refractory cases. Stent placement in transverse sinuses with stenoses has also been proposed as a therapeutic option in selected cases^(10)^, as has ventricular bypass. In cases treated with ventricular bypass, it is important that the radiologist is accustomed to identifying manifestations related to low CSF pressure and its complications, which can occur after the procedure^(11)^.

Although IIH is a rare syndrome, its early diagnosis and treatment are essential to avoid possibly irreversible sequelae. It should also be borne in mind that recurrence is possible, especially in patients who gain weight or become pregnant. It is essential that cases are managed by a multidisciplinary team composed of neurologists, ophthalmologists, and neurosurgeons. For the diagnosis, monitoring, and definition of treatment in patients with IIH, ophthalmologic examinations are essential, particularly funduscopic examination and a visual field test, as well as, in selected cases, optical coherence tomography, ocular ultrasound, fundus autofluorescence, and measurement of visual evoked potentials^(2)^.

## CONCLUSION

Neuroimaging plays an important role in the assessment of IIH, a syndrome that requires rapid diagnosis in order to avoid serious sequelae, especially visual loss. The use of MRI, ideally with venous MRA, can facilitate the diagnosis by excluding cerebral venous thrombosis in patients with other IIH findings.
